# Reproductive health outcomes: Insights from experts and verbal autopsies

**DOI:** 10.4102/curationis.v42i1.1997

**Published:** 2019-09-19

**Authors:** Rose Mmusi-Phetoe, Gloria Thupayagale-Tshweneagae, Oluwaseyi A. Akpor

**Affiliations:** 1 Department of Health Studies, University of South Africa, Pretoria, South Africa

**Keywords:** maternal health, quality care, reproductive health, poor reproductive outcomes, universal access

## Abstract

**Background:**

Reproductive health outcomes are a measure of maternal and neonatal health. South Africa’s state of maternal health is of particular concern because of the two Millennium Development Goals (MDGs) targets for monitoring maternal health, namely MDG 5a, to reduce the maternal mortality rate by three-quarters, and MDG 5b, to achieve universal access to reproductive health by 2015. Maternal mortality ratio and universal access to reproductive health receive unequal responsiveness from government. Monitoring the maternal mortality ratio has received favourable attention compared to ensuring universal access to reproductive health, hence the limited published research findings on the latter.

**Objectives:**

The purpose of this article is to report on the insights from reproductive health experts and verbal autopsies on the determinants of poor reproductive health outcomes.

**Method:**

Individual interviews with a purposively selected sample of six reproductive health experts were conducted, augmented by verbal autopsies of 12 next of kin of women and newborn babies who died within the previous 2 years period of the study. Burnard’s (1995) approach of content analysis was used to analyse the data.

**Results:**

The findings revealed lack of empowerment, inaccessible reproductive health services and separation of patients living with human immune deficiency virus and those patients diagnosed with acquired immune deficiency syndrome.

**Conclusion:**

To meet the reproductive health needs, especially of the rural population, urgent attention is needed to reduce their vulnerability to the risks of poor reproductive outcomes.

## Background

Reproductive health was promoted in the 1994 International Conference on Population and Development (ICPD), which was coordinated by the United Nations. The conference called for a comprehensive approach to sexual and reproductive health (SRH) and reproductive rights (United Nations Programme of Action 1994). The 1994 ICPD argued for the design of policies, programmes and services for reproductive health care that are shaped by a concern for quality care and not the demographic objectives. The same conference (1994 ICPD) signalled a paradigm shift in the field of human reproduction (United Nations Population Fund 2014).

The International Conference on Human Rights, which was held in 2013, namely the ICPD Beyond 2014 conference, reaffirmed a call for a comprehensive approach to SRH and reproductive rights. The ICPD Beyond 2014 conference further proposed that SRH programmes and services be guided by the reproductive health needs and ensure that the human rights of individuals, especially of women, are protected (United Nations Framework of Actions 2014).

The ICPD and the Programmes of Action (POA) conferences recommended that programmes be put in place to ensure that every woman has access to quality reproductive health care (United Nations Population Fund 2014). The five core components of quality reproductive health care are improvement of antenatal, perinatal, postpartum and newborn care; provision of high-quality services for family planning including infertility services; elimination of unsafe abortions; prevention and treatment of sexually transmitted infections, including human immune deficiency (HIV), reproductive tract infections, cervical cancer and other gynaecological morbidities; and promotion of healthy sexuality (World Health Organization 2011).

World Health Organization (2015) maintains that the Millennium Declaration, which articulated eight goals referred to as the Millennium Development Goals (MDGs), promoted reproductive health. The MDG 5 was to be measured by two targets, in which the first target proposed reduction of the maternal mortality rate (MMR) by three-quarters between 1990 and 2015, and the second target called for ensuring universal access to reproductive health between 1990 and 2015.

Monitoring the progress towards achieving MDG 5 has concentrated on the reduction of MMR, and neglected ensuring universal access to reproductive health (Galati 2015). The 2014 combined report of World Health Organization (WHO), United Nations Children’s Fund (UNICEF), United Nations Fund for Population Activities (UNFPA), World Bank and the United Nations Population Division report indicated that progress in reducing the MMR in member countries has been sluggish and uneven. However, the report was silent on the progress made in the target of ensuring universal access to reproductive health (United Nations Framework of Actions 2014). The MMR target therefore seems to have been chosen as a valued outcome to improvements in maternal health compared to provision of universal access to reproductive health. Galati (2015) further indicates that universal access to reproductive health is the most off-track of all the MDGs. The ICPD Programmes of Action (+ MDG action) suffer poor implementation in most developing countries because of declining or collapsed health services resulting in worsening conditions that determine women’s health overall (United Nations Population Fund 2013).

World Health Organization (2014) noted that an estimated 22 million unsafe abortions are experienced globally on a yearly basis, particularly in developing countries. Ngwena and Durojane (2014) added that the sub-Saharan region continues to experience challenges to accessing reproductive health services. The lack of access to these services often leads to increased morbidity and mortality attributable to unsafe abortions, lack of access to obstetric care and the persistence of pandemic levels of HIV infections – to name but a few.

By 2015, South Africa had not achieved much in ensuring universal access to reproductive health care. The contraceptive prevalence rate was 50.2% against the MDG target of 100% in 2003 (Statistics South Africa 2015). The adolescent birth rate was 13.7% in 2011 and the unmet need for family planning was 13.8% in 2008. There were no targets specified or new data available for the last two indicators.

In 2005, the Council of the European Union cautioned that the MDGs could not be attained without progress in achieving the ICPD goal of universal access to SRH (Council of the European Union 2004). Universal access to reproductive health is a prerequisite and key to improving maternal health, reducing maternal mortality, preventing unwanted pregnancies and curbing the spread of sexually transmitted infections, including HIV and acquired immune deficiency syndrome (AIDS).

The ICPD Beyond 2014 International Conference on Human Rights pointed out that MDG 5 suffered a number of challenges in three broad areas, namely those of equality, quality and accountability, and that this needed attention (United Nations Population Fund 2013). In addressing these challenges, the ICPD conference PoA proposed that equality be tackled by reaching out to all people including the marginalised groups; that quality be ensured through making available and accessible, acceptable quality services by providing education, information and services at an affordable cost and managing the provider attitudes that curtail young people’s access to services. Accountability should be addressed by identifying key actors and making governments accountable (United Nations Population Fund 2013).

The United Nations Population Fund (2013) added that maternal health continues to be a global development priority and that the sexual and reproductive health rights (SRHR) agenda should be a key feature of the next development goals, in the ‘post-Millennium Development Goals’ era. Galati (2015) argues that the sustainable development goals (SDGs) agenda builds upon the unfinished work of the MDGs. The researcher further highlighted that enhancing the fundamental aspects of SRHR, including universal access to reproductive health, was a necessary condition to achieving the SDGs.

The SDG number 3.7 calls for member countries to ensure universal access to SRH care services, including family planning, information and education and the integration of reproductive health into national strategies and programmes by 2020 (WHO 2016). This area of study is an under-researched area and does not seem to be well understood in South Africa (Francis et al. 2018; Slabbert et al. 2017).

## Methods

### Design of the study

A qualitative approach was used in this study. An explorative descriptive design was found to be most appropriate as the study aimed at exploring and describing the views of experts and the verbal autopsies of the next of kin of the mothers and neonates who died because of birth-related circumstances.

### The setting

This study was part of a larger study that looked at the overall health needs of the socially excluded, the deprived and the vulnerable women by exploring factors that influence maternal and child health (M&CH) outcomes. The study was conducted in two of the nine provinces of South Africa, namely Gauteng and KwaZulu-Natal (KZN). The health experts are located in Gauteng province where the National Department of Health is based. Gauteng province is the one of the nine provinces of South Africa and is the smallest of the nine provinces accounting for only 1.5% of the land area (Statistics South Africa 2012). For the purpose of this study, experts referred to people employed in the Department of Health with a higher qualification in reproductive health or with over 5 years of experience in the area of reproductive health and employed as such in the department. The experts also had responsibility in policy development and in overseeing its implementation. However, Gauteng province is highly urbanised and has Pretoria as its administrative capital. The South African health headquarters are also based in Pretoria, and hence all the experts were found in Gauteng province.

The next of kin of the women and neonates who had died in the last 2 years because of birth-related circumstances lived in the rural villages of KZN. KwaZulu-Natal is the second most populous province in South Africa (Statistics SA 2012). The inhabitants of KZN who live in rural areas live below the poverty datum line on less than US$2 a day (Statistics SA 2012). KwaZulu-Natal province has been organised into 11 districts, among which is iLembe, a district chosen as a study site. Of all the districts, iLembe has been most affected by the HIV and AIDS epidemic, with the HIV prevalence rate of 45.9% among the antenatal care women in 2013 (South African Department of Health 2013). iLembe has also been classified as one of the districts that are socially deprived (Boerma 2014).

### Study participants and recruitment

Purposive sampling was used to select six experts in the area of reproductive health in South Africa and 12 next of kin of women and the neonates who had died recently (in the last 2 years) as a result of birth-related circumstances. Six reproductive health experts were recruited through the office of the chief director for women’s affairs. The inclusion criterion for reproductive health experts was that (1) they should have had training in the area of reproductive health, (2) they had worked in the area for 1 year or more and (3) they had knowledge of the health system of South Africa, and were responsible for policy development and overseeing its implementation.

The next of kin were recruited through the assistance of the provisional department of health in KZN who gave the researchers registers to go through and identify would-be participants. Participants were later contacted by telephone and arrangements made for an initial visit; those who agreed to participate were explained the study, its objectives and how it will benefit participants in the end. This explanation was then followed by signing of the consent form or placing a thumb for those who did not know how to read and write. The inclusion criteria for next of kin of women who died in birth-related circumstances were that they should be: (1) husband, boyfriend, mother or sister of a woman of reproductive age who had died in the last 2 years of the time of study. (2) The woman had died while pregnant, giving birth or in the postnatal period, both within and outside public health institutions. (3) The next of kin had been living with the woman in the rural areas of KZN. For the neonates, inclusion criteria were that (1) she or he should be the mother of a newborn who had died or a caregiver of a newborn who died following its mother’s death and (2) the newborn should have died in the last 2 years of the study.

### Data collection

Individual interviews were conducted with experts in their offices as per arrangement made between the first author and the experts. Because of time variations, these interviews took place from January to March 2014. Interviews with significant others or next of kin of the women and children who died were conducted in their homes with prior arrangement of time and date. They also took place between January and March 2014. Individual interviews with reproductive experts all started with a similar statement: ‘Tell me your views about reproductive services in South Africa’. This statement was followed by different probes according to the response. Some of the probes were: ‘What can be done to improve the current status of reproductive services?’ ‘How is the implementation of reproductive services done?’ ‘As an expert in the area of reproductive health, who do you think is responsible for the status of the services?’ For the next of kin, the general statement was also used as: ‘Tell me how you feel about the death of your baby? Mother? Wife etc.’ This question was also followed by probes depending on the answer. Some of the probes were: ‘Do you think her or his life could have been saved?’ ‘What is it that the nurses or doctors could have done?’

### Data analysis

An adaptation of Burnard’s (1995) approach of data analysis was used in this study. Audiotaped interviews were transcribed word for word and coded in a series of stages by the first and second authors. Transcription and coding were made independent of each other. In accordance with Burnard’s approach, the tapes were listened to several times to allow the researchers to get deeper meanings of what was said by the participants. The third author was given four transcripts already performed by the two authors to check for consistency and she agreed with the interpretation of the first and second authors on different tapes. Transcription followed detailed written notes and themes were then developed and categorised.

## Results

### Demographic findings

There were two groups of participants: experts in the area of reproductive health and the next of kin of mothers and neonates who died at birth.

The experts interviewed in the study hold a master’s degree or a postgraduate qualification in the area of reproductive health and they all worked in maternal health or neonatal care. Their ages ranged from 38 to 58 years of age and, as shown in Table 1, they had ample experience of 15–30 years.

**TABLE 1:** The profile of reproductive health experts.

Expert pseudonym	Position held	Years of experience in the field
Malebogo	National cluster manager	26
Rebaone	Director for maternal health care	18
Kelebogile	Public health specialist in neonatal care	26
Realeboga	National programme officer	30
Tebogo	National chairperson for saving babies	15
Rearabilwe	Independent consultant in women’s health	28

Six of the next of kin for neonates were mothers, except for two of them where one was a father because the mother was too distraught to talk about her baby’s death. The fifth participant, Lesego, lost her daughter and her granddaughter at the same time and hence was interviewed for her insights in the death of her daughter and that of her granddaughter.

### Themes generated from the study

Table 2 reflects the main themes and subthemes generated from the study.

**TABLE 2:** Demographic profile of next of kin.

Next of kin pseudonym	Gender	Years after deceased	Relationship with deceased
Tiro	Male	2	Husband
Teko	Male	2	Husband
Tele	Male	3	Brother
Leano	Female	2	Sister
Lesego	Female	2	Mother
Lerotse	Female	3	Mother
Masego	Female	2	Mother
Maitseo	Female	2	Mother
Motsei	Female	3	Mother
Lesego	Female	2	Grandmother
Moropa	Female	2	Mother
Moeti	Male	2	Father

**TABLE 3:** Determinants of poor reproductive health outcomes by themes and subthemes.

Themes	Subthemes
1. Lack of empowerment	1.1 Maternal and child health policies disempowering for women 1.2 Poor reproductive health education 1.3 Social norms are not empowering for women
2. Inaccessible reproductive health and poor quality of care	1.1 Inadequate contraception, limited supplies and choice of contraceptive method 1.2 Unmet need 1.3 Attitude of health care providers and incompetence
3. Separation of HIV and AIDS from reproductive health	-

#### Theme 1: Lack of empowerment

Empowerment relates to the way people control the environment that they find themselves in and being able to make informed decisions. According to the participants in this study, there is lack of empowerment as there was no control over the current circumstances of reproductive health. There were three subthemes under this theme.

**Maternal and child health policies that are disempowering:** The current M&CH policies are disempowering for women emerged as a concern and a factor in determining poor reproductive outcomes. Some interviewees recounted disempowering M&CH policies, as shown in the following excerpts:

‘Despite the country’s constitution and policies that appear relevant, the policies are disempowering, especially to women.’ (Malebogo, National cluster manager, 26 years of experience)

When the participant was asked to elaborate this is what she said:

‘There is indirect disempowerment through inattention to public policies and budgets that do not allow women especially adolescents to enjoy sexual and reproductive health rights.’ (Malebogo, 26 years of experience)

‘As people responsible for implementing this policies if we do not have the necessary budgets and infrastructure there is very little that we can do.’ (Kelebogile, 26 years of experience)

‘Reproductive health services for adolescents are limited to female adolescents and are concentrated in towns, this lead to disempowerment of adolescents in rural areas.’ (Rebaone, 18 years of experience)

**Poor reproductive health education:** Lack of accurate education with women, adolescent or parents keeps them uninformed or ill-informed on sexual matters. According to the experts, another thing is that women and girls make choices that impede their reproductive health due to poor reproductive health education, as expressed in the extract below:

‘There is no system to compel teachers to give reproductive health education for boys and girls.’ (Kelebogile, 26 years of experience)

‘Make sure that all the pregnant mothers who are tested are counselled and educated and put on treatment or therapy.’ (Realeboga, 30 years of experience)

**Social norms are not empowering for women:** Women are still disempowered and subjected to their health being assigned a lower priority. One of the participants described her concerns on the culture of silence which surrounds women about their reproductive ailments and exposes them to the inaccessibility of reproductive health services:

‘The context within which we operate does not provide an easy environment for women to negotiate their sexual health.’ (Rebaone, 18 years of experience)

‘In the ICPD language, sexual violence, lack of choice and sex consumption for material gain are all related. For example, a girl might not be poor but may engage with sugar daddies for sex. This makes them vulnerable.’ (Realeboga, 30 years of experience)

#### Theme 2: Inaccessible reproductive health services and unmet need for quality of care

Inaccessible reproductive health services presented itself through inadequate and limited supplies of contraception, influencing the choice of the method, unmet need and the poor interpersonal relations and incompetence of health professionals.

**Inadequate contraception:** Several participants confirmed that family planning services are inadequate in South Africa – hence, the country is experiencing high rates of unplanned pregnancies. The participants were of the opinion that a lower rate of pregnancy would result in a lower incidence of maternal illnesses and deaths; hence, women need to be supported with provision of safe contraception and enhanced coverage of family planning services. An extract below depicts a comment from one expert:

‘The contraceptives that were there 10 years back are no longer available. In the family planning, there is a smaller range of contraceptives compared to 10 years back.’ (Kelebogile, 26 years of experience)

‘Women do not have any choice of contraceptives as they are very few and if a woman is uncomfortable with an injection and the pill shew would not have any other choice as use of a condom is the prerogative of men Female condoms are unpleasant to the eye and hence not an option.’ (Rearabilwe, 15 years of experience)

**Unmet need:** Improved access to quality reproductive services is an essential need in the area of reproductive health. In this study, inaccessibility of quality reproductive services meant that there exists an unmet need for reproductive health services and needs. A positive association was established between the attitude of health professionals, poor-quality health care and women who are young, poor or living in the rural areas. The following extracts confirm poor reproductive outcomes because of the unmet need for family planning. Unmet need in this study also meant poor response and poor-quality care during labour and post-partum. It was reported that the nurses abuse women who are admitted for childbirth verbally; hence, many prefer to deliver at home without professional supervision, and they are thus exposed to many health risks. Patients who are in the labour ward also do not receive continuous monitoring as exemplified by the extracts below:

‘My daughter went for an injection at the clinic. She was given Depo Provera (a long-acting hormonal contraceptive injection), but then she was spotting and bleeding continuously. She went back to report it, but was told that it is normal for a woman to experience bleeding when on the injection. Due to continuous bleeding she stopped using contraception and fell pregnant.’ (Lerotse, mother of deceased)

‘The nurses neglected my 16-year-old daughter’s post-caesarean section because she was young. My daughter experienced difficulties alone after the operation, and died.’ **(**Lesego, mother of deceased teenager)

**Attitude of health care professionals and incompetence:** Limited access, low use of health care services, poor quality of health and consequent high levels of reproductive ailments have been associated with the poor interpersonal relations and attitudes of the health workers, who are often incompetent.

Dissatisfaction about the poor quality of care in the health facilities, the attitude and incompetence of health professionals was further expressed in the following extracts:

‘My sister was 7 months pregnant but had not attended an ANC. She complained about the attitude of the nurses, hence she thought she would only attend the ANC when she was closer to delivering the baby. The hospital is also far and there are no taxis this side of the village because of the mountains. The ambulance was called but it took five hours to arrive. My sister delivered a baby in the toilet, by herself. She died on day 2 at the hospital.’ (Leano, sister of deceased)

‘My baby died at the hands of the nurses. I called the nurses but they did not come.’ (Masego, mother of deceased)

#### Theme 3: Separation of HIV and AIDS from reproductive health

Although the ICPD conferences (1994 and 2013) had indicated that HIV was one of the essential components of reproductive health, integrated SRH services, on the one hand, and HIV prevention, treatment and care, on the other hand, are still treated as separate entities in South Africa. Three of the participants felt that the almost exclusive focus on HIV and AIDS has left reproductive health care services for women in a crisis. One participant commented:

‘While it is fantastic that this government is finally taking AIDS seriously, what we see now is an exclusive focus on AIDS and condoms, without talking about the reproductive health dimension.’ (Kelebogile, 26 years of experience)

Another participant commented that: HIV and AIDS has been taken out of reproductive health, and it needs to be dealt with:

‘The country needs to talk about AIDS and reproductive health and reproductive rights as a combined issue; there is an exclusive focus on AIDS without talking about reproductive health.’ (Tebogo, 28 years of experience)

## Discussion

The factors that determine poor reproductive outcomes in South Africa were examined by means of in-depth interviews with the experts working in the field of reproductive health in South Africa. A lack of empowerment of women followed by inaccessible reproductive health services and lastly separation of HIV and AIDS from reproductive health surfaced as key themes and factors determining poor reproductive outcomes.

According to Alimoradi et al. (2017), empowerment is key in health promotion and the health-related quality of life. Empowerment allows one to attain control over their health through the decisions that they make (Phuti 2018). The empowerment process within the gender and development context seeks to address the powerlessness of women and has the greatest potential of reducing reproductive ill health and mortality. The 1994 ICPD PoA recognises the empowerment and autonomy of women and the improvement of their political, social, economic and health status as a highly important end in itself (United Nations Framework of Action 2014).

All six experts in this study viewed the lack of empowerment of women as contributing to the inaccessibility of reproductive health and consequently the poor reproductive outcomes. The M&CH policies and services are presented as a first subtheme under the theme of lack of empowerment. Policies that do not seem to be explicit on M&CH stood out as disempowering for women and were viewed as a serious concern which subjected women to ill health and even death through marginalising them in a given context.

Poor reproductive health education emerged as a second subtheme under the theme of lack of empowerment. In the context of empowerment, more information would ensure that choices made regarding contraception are informed within an expanding framework of information, knowledge and an analysis of options available. The experts highlighted that although the national M&CH policy indicates the importance of health education to influence health perceptions and behaviours, they actually remain silent on how they would achieve it, thus still not advancing beyond the level of rhetoric. The delivery of a reproductive health education in the provinces has suffered as a result.

The social norms that are not empowering to women form the third point under the theme of lack of empowerment. The experts felt that the social norms disabled transformative change that would empower women to shape their own lives. The social norms manifest in unequal access to education and health and deepen feminine poverty. The social norms are holding women back and hinder their empowerment. Women and girls are unable to exercise choice over their sexual and reproductive integrity if they are not empowered to make an informed choice within an expanding framework of information, knowledge and analysis of options available, as a result of social norms (United Nations Population Fund 2014).

In line with Stein’s (1997) description of empowerment, the lack of empowerment of women is channelled through many paths until the woman dies from direct causes such as maternal anaemia, pregnancy hypertensive disorders and infections. Stein further argues that there is a relationship between health, empowerment and self-determination of women (Stein 1997).

Inaccessible reproductive health services that are characterised by poor reproductive health service provision, inadequate contraception, unmet needs as well as negative professional attitudes emerged as a factor in determining poor reproductive outcomes. Historically, reproductive health care in South Africa was delivered through family planning units, although the emphasis was on the provision of contraception for reducing fertility (Patel 2014). The participants highlighted that inaccessibility to and poor reproductive health currently presents itself through inadequate and limited choice in contraception; the services that are still provided occur through narrow vertical programmes in the inadequately equipped public infrastructure, equipment and workforce; poor relations between the providers and recipients; and women’s continuously unmet reproductive health needs.

United Nations Fund for Population Activity defines the unmet need for family planning as a gap between women’s reproductive intentions and their contraceptive behaviour. Unmet women’s reproductive health needs surfaced through the low-quality care services, side effects and inconveniences of contraceptive methods and the negative attitude of health care providers. Van Lith, Yahner and Bakamjian (2013) indicated that 8 million women in sub-Saharan Africa have an unmet need for contraception. In their study in Malawi, Habte and Namasusu (2015) reflected that women living with HIV experience increased levels of unmet needs in the form of limited access to family planning and reproductive health services than the general population. Contraception should form part of every reproductive health programme as it is key to addressing maternal mortality associated with unplanned pregnancy (Patel 2014).

Limited access to reproductive health care was also associated with poor interpersonal relations and attitudes of health workers. Women come into contact with unsympathetic and insensitive health care providers in health care centres. The negative attitude is mostly experienced by the poor, rural and black women in South Africa, while the elite black and white women are served well. Attitudes impact on the quality of health care. In their study in Africa, Mannava et al. (2015) confirmed that the negative attitude and behaviours of health care providers undermined health care seeking and access to reproductive health.

The study found that inadequate contraception, unmet needs and poor-quality reproductive health services, especially in the rural areas, bring about different reproductive health outcomes as a result of differences in accessing reproductive health services. The study further established that victims of HIV and AIDS were dealt with as if they were different from reproductive health patients. The economically and socially underprivileged black rural women are the most vulnerable and disempowered groups in protecting themselves against unplanned pregnancies and against HIV and AIDS, because of inaccessible services, limited education and inadequate access to accurate information on sexuality, contraception and reproduction. In the study on the HIV epidemic and SRH policy integration conducted by Cooper et al. (2015), policy development and implementation of SRH and HIV (SRH-HIV) services were found to be deficient in South Africa. The authors’ further highlight that the participants in that study, mostly policymakers, supported SRH-HIV-integrated policy and services.

## Conclusion

The study established that poor focus on reproductive health and inattention to reproductive health needs expose women to the risk of reproductive ill health, maternal morbidity and the associated mortality. Hence, recognition of and ensuring access to reproductive health, and meeting reproductive health needs are prerequisites to reduction of the maternal mortality rates. The factors determining poor reproductive health outcomes further provide an illustration of the shortcomings in the current M&CH and family planning approaches and interventions. The empowerment of women by creating awareness of the available reproductive health care services could be ensured through an integrated approach and inter-sectoral approaches. The day-to-day reproductive health needs could be ensured through the provision of skilled and caring health care professionals as well as sufficient budget thus making accessible to all equitable and quality reproductive health care services.
